# Comparable choroidal thickness between treated eyes and untreated fellow-eyes in patients with unilateral neovascular AMD: a paired-eyes comparative study

**DOI:** 10.1007/s00417-025-06751-7

**Published:** 2025-03-08

**Authors:** Francesco Cinque, Femke M van den Tillaert, Suzanne Yzer, Anita de Breuk, Tom J Heesterbeek, Carel B Hoyng, Yara TE Lechanteur

**Affiliations:** Department of Ophthalmology, Donders Institute for Brain, Cognition and Behaviour, Radboud University Medical Center, Geert Grooteplein Zuid 10, Nijmegen, 6525 The Netherlands

**Keywords:** Choroid, Macula, Anti-VEGF, Age-related macular degeneration

## Abstract

**Aims:**

To investigate the potential effect of anti-VEGF treatment on choroidal thickness (CT) in unilateral neovascular age-related macular degeneration (AMD) patients.

**Method:**

This is a cross-sectional study where patients were included as part of an ongoing prospective study which included patients with unilateral neovascular (n) AMD. The fellow-eye served as control. All patients had spectral-domain optical coherence tomography (SD-OCT) with enhanced depth imaging (EDI) done at every visit. CT was measured independently by two graders at five locations: subfoveal, 1500 micron temporal and nasal, 3000 micron temporal and nasal. The average of the measurements was used after statistical verification of their accuracy. CT differences were initially analysed via a paired T-test and later via multiple linear regression. Variables such as number of injections were studied and presence of geographic atrophy (GA) in fellow-eyes was evaluated via SD-OCT.

**Results:**

A total of 112 patients met the inclusion criteria (Female 67%). The median (IQR) years of treatment was 2.6 (4.1). The subfoveal choroidal thickness (SFCT) in the neovascular (NV) eye appeared thinner in the NNV eye initially (-11.0 μm difference between NV and NNV SFCT (CI -23.4 to 1.3). However, after age-adjustment this trend disappeared (CI -29.8 to 4.6). In fact, apart from age (CI -6.2 to -0.1)), no other variable including number of anti-VEGF injections (CI -1.5 to 1.4) predicted SFCT. Presence of GA in fellow eyes did not influence the SFCT compared to non-GA fellow eyes, difference (CI -59.7 to 46.6).

**Conclusions:**

This study shows no statistically significant CT difference in NV versus NNV eyes. There was no relationship between number of injections and CT.

**Key Messages:**

***What is known***

- Intravitreal injection with anti-vascular endothelial growth factors (anti-VEGF) is the mainstay treatment for exudation secondary to neovascular AMD. One quarter of anti-VEGF treated neovascular AMD patients will develop signs of macular atrophy within 2 years, possibly related to anti-VEGF treatment.

***What this study adds***

- A hypothesized mechanism for atrophy induction is the effect of anti-VEGF on choroidal thickness. In this cross-sectional study, we found a non-significant 11 micron difference between anti-VEGF treated eyes and non-treated eyes in long-term follow-up neovascular AMD patients. A relationship between choroidal thinning and the number of anti-VEGF injections was furthermore not shown.

***How this study might affect research, practice or policy***

- There is no significant choroidal thickness difference between anti-VEGF treated and non-treated long-term follow-up neovascular AMD. We therefore suggest that atrophy induction through choroidal thinning secondary to anti-VEGF injections is of limited concern.

**Supplementary Information:**

The online version contains supplementary material available at 10.1007/s00417-025-06751-7.

## Introduction

Age-related macular degeneration (AMD) is a progressive, neurodegenerative macular disease and the leading cause of visual impairment in the Western world [1]. Intravitreal injection with anti-vascular endothelial growth factors (anti-VEGF) is the mainstay treatment for exudation secondary to neovascular AMD. It is current practice in the western world to aim for a fluid-free optical-coherence tomography (OCT) image which has also resulted in a substantial treatment burden [2]. There is a high risk of disease-recurrence if anti-VEGF treatment is discontinued [3]. Considering our ageing population, the number of patients that receive continuous anti-VEGF injections will continue to grow [1]. Concerns have arisen as to whether chronic anti-VEGF treatment increases the risk of secondary macular atrophy [4–6]. 

In the CATT-study, the percentage of patients with macular atrophy increased from 17 at year two to 38 at year five [7]. In the IVAN trial, a higher number of eyes with macular atrophy was observed in the monthly treated group compared to the as needed group (34% vs. 26% *P* = .03) [8]. Overall one-quarter of neovascular AMD patients treated with various anti-VEGF developed macular atrophy within 1 to 2 years following treatment initiation [4]. Choroidal thinning is one of the main proposed mechanisms through which macular atrophy might be induced [4]. All commonly used anti-VEGF have been associated with decreasing of CT, particularly during the loading phase [9–14]. Koizumi, et al. for example reported a decrease of 35.8 μm in eyes receiving aflibercept treatment after 12 months [10]. There is limited evidence that choroidal thinning persists after long-term anti-VEGF treatment. One notable exception is Govetto, et al. who reported that choroids in NV-eyes were thinner than their fellow-eye counterpart (subfoveal: 200.69 micron vs. 184.36 micron *P* = .02) [15]. 

Controversy with regard to the effect of anti-VEGF treatment on CT remains; in a recent large, prospective study there was no association with ranibizumab treatment and macular atrophy [16]. Moreover, a small pilot study of 10 patients with geographic atrophy with secondary macular neovascularisation did not observe an increase of geographic atrophy enlargement post-treatment [17]. 

This study investigates the effect of anti-VEGF injections on CT and specifically addresses the potential concerns over long-term treatment with anti-VEGF for NV-AMD by comparing CT between these unilateral treated eyes and the fellow non-neovascular (NNV) AMD eyes.

## Method

### Design

Data was collected from Jan 2018 to jan 2022 as part of a prospective study at Radboud university medical centre. In this cross-sectional study, we measured choroidal thickness (CT) at baseline visit. All patients had neovascular AMD in one eye which was treated with, at minimum, 3 non-specified anti-VEGF injections. Exclusion criteria included previous photodynamic therapy, pathological myopia, advanced glaucoma, or other retinal diseases such as diabetic retinopathy that could interfere with the diagnosis of AMD. Patients were subjected to a comprehensive dilated eye-exam that included extensive imaging such as Spectral-Domain Optical Coherence Tomography (SD-OCT) with Enhanced Depth Imaging (EDI) [SpectralisTM HRA + OCT (Heidelberg Engineering, Heidelberg, Germany)]. In addition, visual acuity (VA) was measured using the Early Treatment Diabetic Retinopathy (ETDRS) letter chart. Ophthalmic history and medical history were assessed using a questionnaire. Ophthalmic history includes the time since first eye involvement to baseline and the number and type of anti-VEGF drugs. This study was conducted in accordance with the tenets of the Declaration of Helsinki and was approved by the local ethics committee of the Radboud university medical centre, Nijmegen, The Netherlands. All participants provided written informed consent prior to participating.

### Grading

CT was measured independently by two graders (FC) and (FT) using the calliper function in Heidelberg. CT was measured from BM to the outer border of the suprachoroid, subfoveally as well as 1500 and 3000 micron in the temporal, and nasal direction [18]. Measurement agreement was statistically analysed using Intraclass Coefficients Correlations (ICC). We used the mean of the graders as our final CT approximation. To increase measurement accuracy for subfoveal measurements, we additionally used Bland-Altman plots to visualise outliers (defined as exceeding a measurement discrepancy of two standard deviation [SD] from the mean SFCT). Outliers were revalued and corrected by experienced retinal specialists (SY and YL). Corrected values were then used for further analyses instead of the mean of the two graders. In addition, the Bland-Altman plots were used to assess the proportionality of measurement discrepancy and hence grader bias. Lastly, OCTs were graded by FC for the presence of complete retinal pigment epithelium and outer retinal atrophy (cRORA) as defined according to the consensus definition of atrophy associated with AMD [19]. 

### Outcome and statistical analysis

We used a two-way random effects model for the ICC. We also computed a one-sample T-test for the mean difference of the measurement discrepancies to statistically assess grader bias and report the SD of the measurement discrepancies. Our main outcome is the difference in CT between NV and NNV fellow-eyes which was assessed via a paired samples T-test. We also performed an age-corrected SFCT analysis using multiple linear regression. In addition, we modelled the influence of various clinical predictors on SFCT. For SFCT of NV eyes we investigated the relationship between SFCT (dependent variable) and, age, sex, visual acuity, cRORA, smoking, number of injections, type of anti-VEGF drug(s) (bevacizumab, ranibizumab, aflibercept), as independent variables using multiple linear regression. Similarly, for NNV eyes we performed multiple linear regression with SFCT as dependent variable and age, sex, visual acuity, cRORA, smoking as independent variables. We report adjusted R^2^ as model performance. *P* values of < 0.05 were considered statistically significant and all statistical analyses were performed using IBM SPSS Statistics for Windows, version 24 (IBM Corp., Armonk, N.Y., USA).

## Results

### Baseline

In total 116 patients met the criteria. However, in 4 cases the quality of the EDI-OCT was too weak to allow visualisation of the sclero-choroidal junction and thus these cases were excluded. Baseline characteristics of the remaining 112 patients are shown in Table (1).

**Table 1:** Baseline characteristics

Characteristics	Value *N* = 112
Time since first eye involvement, median (IQR), range, yrs	2.9 (4.8) 0 to 20
Age, m (SD), years	74.4 (8.2)
Female, No. (%)	75 (67%)
Caucasian, No. (%)	112(100%)
Visual acuity NV eye, m (SD), logMAR	0.50 (0.43)
Visual acuity NNV eye, m (SD), logMAR	0.07 (0.20)
cRORA NV eye, No. (%)	24/112 (21%)
cRORA NNV eye, No. (%)	46/112 (41%)
Supplement use, No. (%)	77 (69%)
Months since first eye developed MNV, median (IQR) range	35 (57) 0–245
Number of injections, median (IQR) range*	13 (23) 3–120
Type of injections, No. (%)	
One type of anti-VEGF	41
Bevacizumab	36 (88%)
Aflibercept	1 (2%)
Ranibizumab	4 (10%)
Combination of anti-VEGF medications	49
Bevacizumab + Aflibercept	21 (49%)
Bevacizumab + Ranibizumab	7 (14%)
Bevacizumab + Aflibercept + Ranibizumab	18 (37%)
Unknown type of injections	22

*NV* Neovascular, *NNV* non-neovascular, *cRORA* complete retinal pigment epithelium and outer 440 retinal atrophy, *MNV* macular neovascularisation. *n = 10 did not provide details about number of past injections,

Mean age (SD) was 74.2 (8.3) years. The median (IQR) time since first-eye involvement with neovascular AMD was 2.9 (4.8) and ranged from 0 to 20 years. The median (interquartile range, IQR) number of injections received was 13 (23) during the median (IQR) 2.6 (4.1) years of treatment. Baseline visual acuity 

### Evaluating CT differences

(SD) was 0.50 (0.4) logMAR for NV eyes and 0.08 (0.2) logMAR) for NNV eyes. Of the 41 patients who were treated with one type of anti-VEGF, the majority (88%) was treated with bevacizumab. The 49 patients who were treated with a combination of anti-VEGF medications were most often treated with a combination of bevacizumab and aflibercept (49%) (Table 1).

### Grader agreement

The lowest ICC was measured at the 3000 micron temporal location (0.941 NV and 0.944 in the NNV). Subfoveal ICC’s were 0.991 and 0.993 for NV and NNV eyes. The other ICC’s are reported in online resource Table (1). Bland-Altman plots showed approximately symmetrical measurement discrepancies, therefore not suggesting bias of either grader (see online resource Fig. 1). Statistical analysis confirmed this, as the mean (SD) difference of the measurement discrepancies was 0.0 μm (20.1) (*P* = 1) µm and 2.5 μm (18.6) (*P* = .1) for NV and NNV eyes. An example of the CT measurement as performed by the graders is visible in online resource Fig.  2.

### Evaluating CT differences

**Fig. 1:**
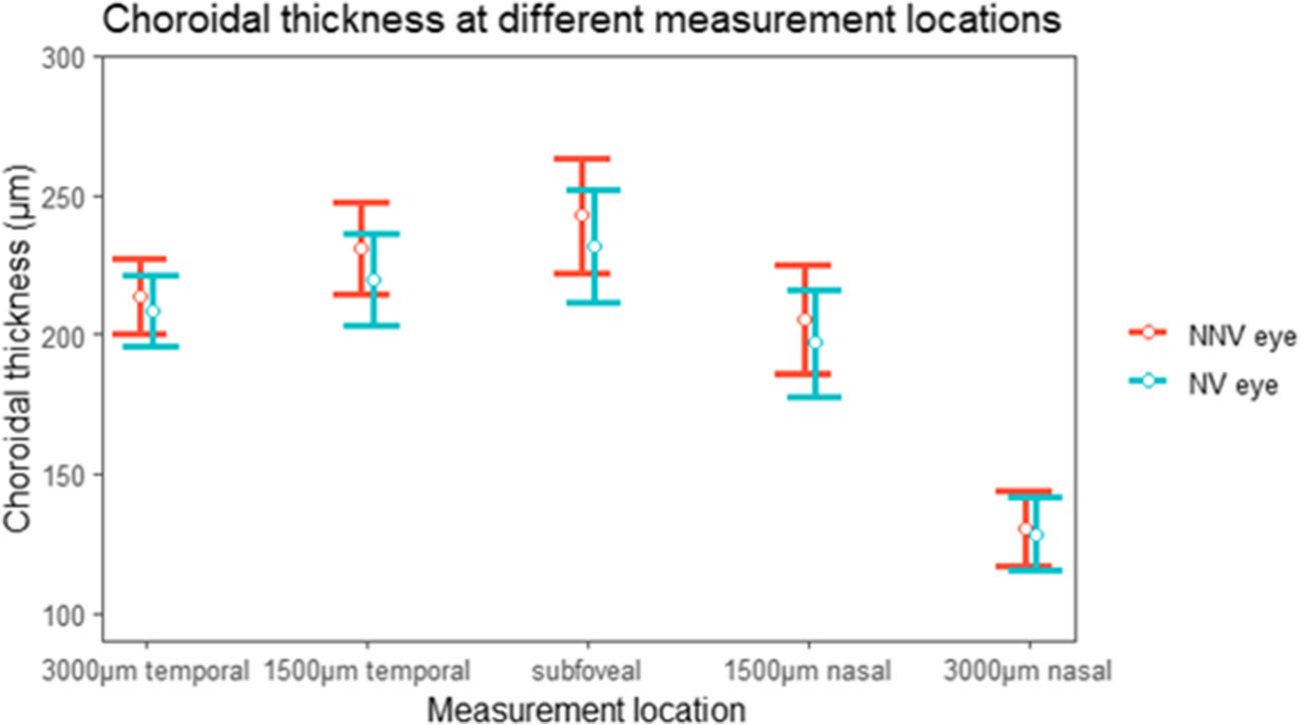
Choroidal thickness in neovascular eyes and non-neovascular eyes Fig (1) shows the choroidal thickness at all measurement locations for neovascular (NV) and non-neovascular (NNV) eyes. The error bars represent the 95% confidence interval

**Fig. 2:**
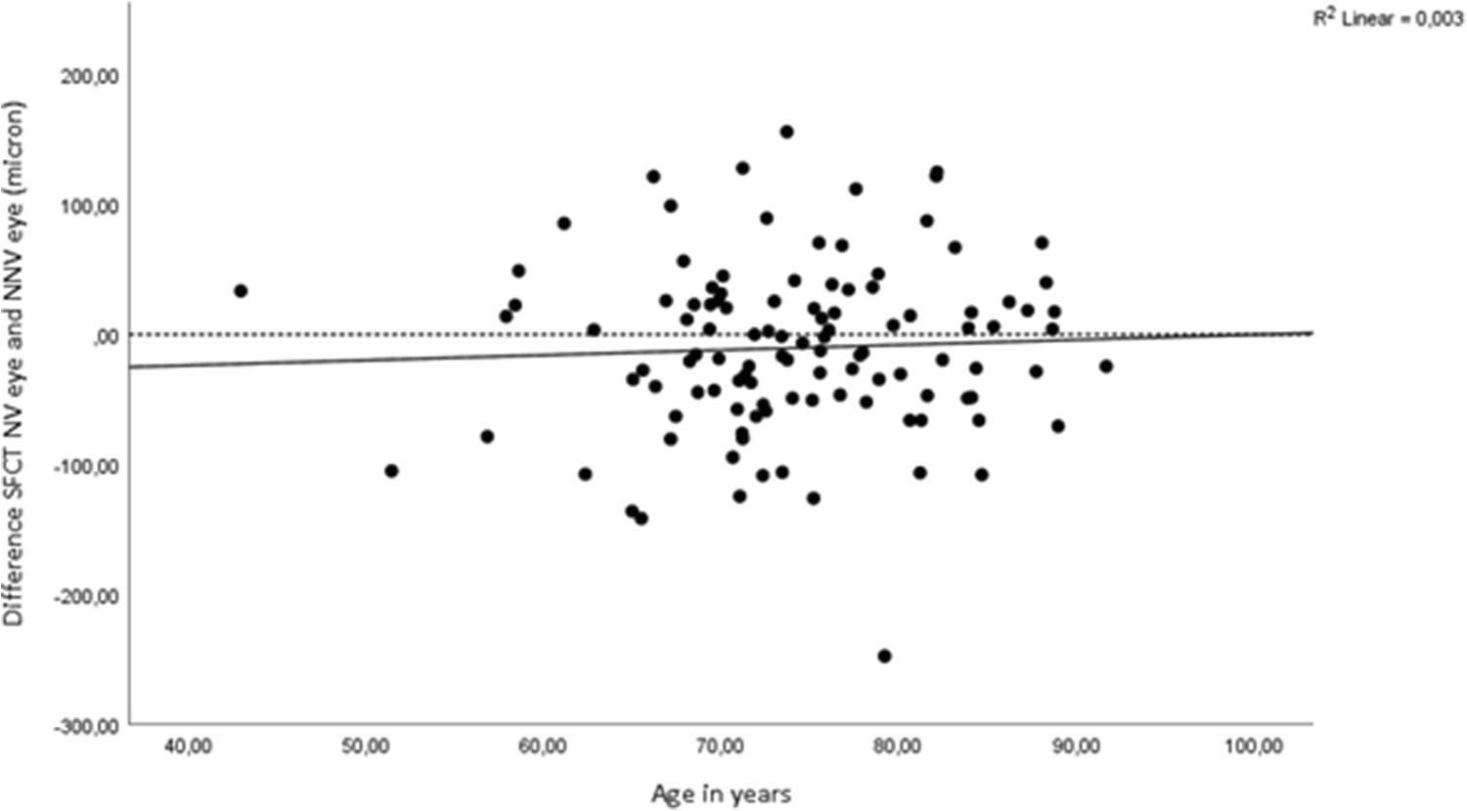
The difference between subfoveal choroidal thickness and age SFCT subfoveal choroidal thickness. The SFCT thickness difference for NV and NNV eyes (y-axis), and age (x-axis)

The SFCT difference between NV and NNV eyes was 11 μm (*P* = .08) (CI −23.4 to 1.3) as shown in Table (2) and Fig. (1). CT seemed thinner in the NV eye at every location, however no difference reached statistical significance. After correction for age, the CI for the difference between NV and NNV eyes widened of the CI (CI −29.8 to 4.6) (*P =* .15). Also, we checked trends on the effect of age on the difference between NV and NNV SFCT and observed that the difference between NV and NNV SFCT diminished with advancing age (see Fig. (2)).

**Table 2:** Choroidal thickness at every Measurement Location

Location	Mean CT NV eye (SD) µm	Mean CT NNV eye (SD) µm	Difference in µm (*P* value)	CI
Subfoveal	231.7 (109.3)	242.7 (110.3)	−11.0 (0.08)	−23.4 to 1.3
1500 micron nasal	197.2 (103.0)	206.7 (105.9)	−9.5 (0.1)	−21.2 to 2.1
3000 micron nasal	127.5 (70.1)	130.1 (72.7)	−2.6 (0.5)	−10.6 to 5.3
1500 micron temporal	220.2 (89.1)	229.7 (87.0)	−9.4 (0.1)	−21.1 to 2.2
3000 micron temporal	208.9 (67.3)	214.5 (70.7)	−5.6 (0.3)	15.7 to 4.5

### Clinical predictors of SFCT

For NV and NNV eyes, age had a modest effect on SFCT (CI −5.9 to − 0.4) (*P* = .02) (CI −6.5 to −1.2) (*P* = .01) (Table 3). All other independent variables failed to predict SFCT for NV and NNV eyes. Adjusted R^2^ was 0.06 and 0.06 for NV and NNV eyes. We visualised the relation of age and SFCT for NV and NNV eyes in online resource Fig. (3) (R^2^ was 0.08 and 0.07 for NV and NNV eyes). Neither model violated any of the following statistical assumptions: linear relation of independent and independent variables, normality of residuals, absence of multicollinearity.

**Table 3:** Clinical predictors of SFCT in the neovascular eye

	SFCT NV	SFCT NV				SFCT NNV	
*Independent variables*	*Estimates*	*CI*		*P*	*Estimates*	*CI*	*P*
(Intercept)	387.0				496,2		
Age	−3,2	−5.9 to −0.4		0.02*	−3,8	−6.5 to −1.2	0.01*
Sex	50.0	−1.6 to 96.0		0.06	17,5	−29.2 to 64.1	0.5
Visual acuity (logMAR)	−42.4	−86.0 to 34.0		0.4	−14,2	−114.6 to 86.1	0.8
cRORA	−7.9	−60.0 to 44.4		0.8	7,7	−43.5 to 59.0	0.8
Smoking (ever/current)	23.3	−26.1 to 71.0		0.4	−0,7	−47.4 to 46.0	0.9
Year since first injection	5.4	−3.5 to 11.2		0.3	4,6	−0.6 to 9.8	0.1
Number of injections	−0.6	−1.4 to 1.3		0.9			
One type of anti-VEGF		−65.4 to 54.1		0.9			
Combination anti-VEGF		−81.6 to 48.6		0.6			
Adjusted R^2^		0.06				0.06	

*indicates statistical significance. SFCT subfoveal choroidal thickness, cRORA complete retinal 454 pigment epithelium and outer retinal atrophy.

Table 3 shows estimates, CI and *P* values from two linear regression model with SFCT of NV eye and SFCT of NNV eye as dependent variable. We used pairwise deletion for our estimates.

## Discussion

### Main findings

In this cross-sectional paired eye study using data from a prospective cohort study, we observed a statistically insignificant 11 μm difference between SFCT of NV and NNV AMD eyes (*P* = .08). To appreciate this difference, note that the diameter of a human red blood cell is 7.5 to 8.7 μm [20]. Also, CT at other measurement locations in NV and NNV eyes did not differ significantly. In a similar vein, we attempted to elucidate the relationship with various clinical predictors and SFCT. In our analysis, only age had a marginal relationship with SFCT with an R^2^ of 0.08 and 0.07. In fact, for NV SFCT, the combined variance explained of age, sex, visual acuity, cRORA, smoking, number of injections, type of injections: bevacizumab, ranibizumab, aflibercept, amounted to a paltry adjusted R^2^ of 0.06. This selection of predictors represents some of the most cited associations with CT, in particular age [21], yet it leaves 94% of the variance unaccounted for in our analysis, suggesting that CT is either highly heterogenous or poorly understood.

### Are choroids of NV eyes thinner?

Concerning the difference between NV CT and NNV CT we found an 11 micron difference while Govetto et al. reported a fifteen micron difference between SFCT (*P* = .02). One difference between our study and theirs is a power differential (*N* = 161). A second consideration is that the median (IQR) time since first-eye involvement was 2.9 (4.8) years and ranged from 0 to 20 years in our sample while their sample was based on retrospective consecutive diagnoses of neovascular AMD in one eye.

Also, cRORA did not influence SFCT in NV or NNV eyes (NV eyes: *P* = .8)(NNV eyes: *P* = .8) (Table 3). Contradictory reports have emerged regarding disease stage and CT. A large study showed that intermediate AMD was associated with thicker CT while early AMD was not [22]. Govetto et al. reported the largest CT difference in early staged eyes [15]. Eyes with GA seem to have thinner CT [23]. 

Setting the issue of statistical significance aside, how does a 15 or 11 micron difference relate to our overarching hypothesis of CT and atrophy? Indeed, we performed this study since there are arguments that anti-VEGF injections could lead to macular atrophy through CT thinning. Due to the observational cross-sectional nature of our study it is perhaps impossible to attribute potential thinning strictly to anti-VEGF since progression of AMD co-occurs. The relationship between disease progression in neovascular AMD is additionally complex as CT is shown to increase when exudation develops [24, 25]. Similarly, due to high recurrence, most neovascular AMD patients are continuously treated with anti-VEGF [3]. No cross-sectional or longitudinal study could therefore realistically separate the effects of progression of AMD and treatment with anti-VEGF. CT functions thus as an imperfect resultant measure of contrary and conflicting processes that either induce thinning or thickening. To provide definitive evidence of atrophy induction as a result of anti-VEGF treatment would require the unethical application of an anti-VEGF regiment in non-AMD patients. Alternatively, there is no evidence of GA-enlargement secondary to macular neovascularisation development regardless of anti-VEGF treatment intensity [17]. In patients with diabetic macular edema a retrospective analysis of 1437 patients showed that 4% developed a CT of less than 200 microns during the study period and no association with anti-VEGF treatment was found [26]. In sum, considering that our statistically non-significant CT difference was observed in this long-term treated cohort of nAMD patients, it seems unlikely that through persistent choroidal thinning our patients will have been exposed to an increased risk of macular atrophy.

### Anti-VEGF

All the most commonly used anti-VEGF medications have at one point been linked to CT reduction [14]. Though effect sizes vary [27]. In our cohort, the majority of patients treated with a single anti-VEGF have been treated with bevacizumab reflecting current Dutch best-practice (Table 1). Aflibercept in particular seems to bring about a clear effect as evinced by Koizumi et al. who reported a 35.8 μm decrease in CT in 12 months in 58 treatment-naïve neovascular AMD patients [10]. Of note, whilst our cohort did not feature treatment-naïve neovascular AMD patients, the majority of patients (86%) have at some point been treated with aflibercept (86%).(Table 1) Since no such CT diminution appears to have persisted in our cohort, perhaps aflibercepts effect on CT is limited to the loading phase [28]. Further complicating the comparison of these findings, apart from differing methodologies, are the many factors potentially modifying CT. These include but are not limited to: diurnal rhythm [29] intra-ocular pressure [30], axial length [31], certain medications [32], and coffee intake [33]. 

### Strengths and weaknesses

A major strength of our design is the cross-sectional within-patient comparison of both eyes as we thus control for many of the aforementioned factors that seem to influence CT. A second major strength is our systematic approach to the measurement of CT. Our mean measurement difference of 0 and 2 μm ((*P* = .9)(*P* = .1) for NV and NNV eyes indicates that both graders interpretated choroidal borders similarly and that this variation is due to random fluctuation rather than systematic bias. By taking the mean of both graders, we hoped to correct for this random fluctuation and provided a good approximation of the true CT. However, we want to remark that the 20.0 and 18.0 μm SD of the mean differences is large in comparison to the effects studied. It seems likely that random error therefore still obfuscates the relations under investigation. Another limitation is the need for sufficient statistical power to identify small effects accurately. This concern extends to potentially fruitful analyses of CT in relation to MNV subtype which have not been performed for this reason. A study of comparable sample size did not find statistically significant differences in CT between MNV subtypes [34]. Additionally, the use of real-world data can hinder the precise disentanglement of certain associations being studied.

### Future research

Moving forward, we welcome the more widespread use of automated choroidal segmentation [35]. Once this technology is demonstrated to clearly outperform humans, even in cases where the sclero-choroidal junction is poorly visible, employing it could aid the expansion of present data-sets without labour intensive human measurement. Automatic measurement would have the added benefit of homogenizing the measurement process which would aid cross-study evaluations. Lastly, local changes in the choriocapillaris have been observed at various AMD-stages and may be further quantified using OCT-angiography [36, 37]. 

## Conclusion

We did not find a statistically significant difference in CT between NV eyes treated with anti-VEGF versus NNV fellow-eyes in a long term nAMD follow-up cohort. We therefore conclude that potentially induced choroidal thinning through the use of necessary anti-VEGF for NVAMD appears to be of limited concern as a risk factor for macular atrophy.

## Supplementary Information

Below is the link to the electronic supplementary material.

ESM 1 (DOCX 15.9 KB)

## Abbreviations

anti-VEGF: Anti-vascular endothelial growth factors
AMD: Age-related macular degeneration
OCT: Optical-coherence tomography
CT: Choroidal thickness
SFCT: Subfoveal choroidal thickness
NV: Neovascular
NNV: Non-neovascular
cRORA: Complete RPE and outer retinal atrophy
GA: Geographic atrophy
