# Supplementary material for: Comparable choroidal thickness between treated eyes and untreated fellow-eyes in patients with unilateral neovascular AMD: a paired-eyes comparative study
Source: Graefes Arch Clin Exp Ophthalmol. 2025 Mar 8;263(6):1543–51. doi: 10.1007/s00417-025-06751-7 (PMC12238156; doi:10.1007/s00417-025-06751-7)
Supplement: Supplementary file 1 — (DOCX 15.9 KB) [file 417_2025_6751_MOESM1_ESM.docx]

**ONLINE RESOURCE TABLES**

**Online resource table (1).** Intraclass Correlation Coefficient

| Location | Intraclass Correlation Coefficient | |
| --- | --- | --- |
|  | NV | NNV |
| Subfoveal | .991 | .993 |
| 1500 µm nasal | .986 | .991 |
| 3000 µm nasal | .971 | .962 |
| 1500 µm temporal | .980 | .981 |
| 3000 µm temporal | .949 | .944 |

ICC intra-class correlation coefficient.

For the ICC we used a two-random effects model, with consistency definition. We report the average measure.

**ONLINE RESOURSE FIGURE TITLES AND LEGENDS**

**Online resource Figure (1).** Bland-Altman plots

SFCT subfoveal choroidal thickness.

Bland-Altman plots showing agreement for subfoveal choroidal thickness between the two graders for NV (A) and NNV (B) eyes. The mean difference between the graders is visualised using a continuous line. The dotted lines represent the limits of agreement of 95% or 1.96 SD that contain 95% of all measurement differences between graders.

**Online resource Figure (2).** Example of choroidal measurement

A. Measurement in non-neovascular eye by grader 1. B. Measurement of neovascular eye grader 1. C. Measurement of non-neovascular eye by grader 2. D. Measurement of neovascular eye grader 2.

**Online Resource Figure (3).** Subfoveal choroidal thickness and age

NV Neovascular, NNV non-neovascular, SFCT subfoveal choroidal thickness.

The SFCT thickness for NV and NNV eyes (y-axis), and age (x-axis).
